# The relationship between non-high-density lipoprotein cholesterol to high-density lipoprotein cholesterol ratio (NHHR) and hyperuricaemia

**DOI:** 10.1186/s12944-024-02171-4

**Published:** 2024-06-21

**Authors:** Zhaoxiang Wang, Menghuan Wu, Ruiqin Du, Fengyan Tang, Mengjiao Xu, Tian Gu, Qichao Yang

**Affiliations:** 1 Department of Endocrinology, Affiliated Kunshan Hospital of Jiangsu University, Kunshan, Jiangsu 215300 China; 2 Department of Cardiology, Xuyi People’s Hospital, Xuyi, Jiangsu 211700 China; 3 Department of Endocrinology, PLA Rocket Force Characteristic Medical Center, Beijing, 100088 China; 4 Department of Endocrinology, Affiliated Wujin Hospital of Jiangsu University, Changzhou, Jiangsu 213017 China; 5 Department of Endocrinology, Wujin Clinical College of Xuzhou Medical University, Changzhou, Jiangsu 213017 China

**Keywords:** Hyperuricaemia, Non-HDL-c, NHHR, Nonlinear relationship, NHANES

## Abstract

**Purpose:**

The ratio of non-high-density lipoprotein cholesterol (non-HDL-c) to high-density lipoprotein cholesterol (HDL-c) (NHHR) is a novel comprehensive lipid index. The aim of this study was to investigate the relationship between the NHHR and the prevalence of hyperuricaemia (HUA) in the adult population of the U.S.

**Methods:**

This cross-sectional study collected data from the National Health and Nutrition Examination Survey (NHANES) (2007–2018). HUA was defined as a serum uric acid (SUA) concentration ≥ 7 mg/dL in men and ≥ 6 mg/dL in women. Multivariate logistic regression models and the restricted cubic spline (RCS) method were applied to examine the relationship between the NHHR and the risk of developing HUA. Subgroup analyses and interaction tests were also performed.

**Results:**

The prevalence of HUA increased with increasing NHHR values (9.01% vs. 13.38% vs. 17.31% vs. 25.79%, *P* < 0.001). The NHHR was independently correlated with the risk of developing HUA (OR = 1.10, 95% CI: 1.05–1.16; *P* < 0.001). Furthermore, the risk of developing HUA was significantly greater among individuals with the highest NHHR quartile than among those with the lowest NHHR quartile (OR = 1.94, 95% CI: 1.62–2.33; *P* < 0.001). This relationship was consistent across subgroups. According to the RCS analysis, an inverted U-shaped relationship existed between the NHHR and the risk of developing HUA.

**Conclusions:**

The NHHR was closely associated with an increased risk of developing HUA. Further studies on the NHHR could be beneficial for preventing and treating HUA.

**Supplementary Information:**

The online version contains supplementary material available at 10.1186/s12944-024-02171-4.

## Introduction

Hyperuricaemia (HUA) is a typical metabolic disorder characterized by an elevated uric acid level in the plasma that exceeds normal limits. HUA not only serves as an early stage indicator and a primary cause of gout but is also considered an important risk factor for the development of cardiovascular diseases, diabetes mellitus, and chronic kidney disease as well as increased mortality rates [1–4]. Currently, the global incidence of HUA is on the rise, imposing a significant burden worldwide [5, 6]. Despite this, its treatment continues to be less than optimal.

Non-high-density lipoprotein cholesterol (non-HDL-c) refers to all potentially atherogenic cholesterol found in various lipoprotein particles, such as low-density lipoprotein cholesterol (LDL-c), lipoprotein (a), intermediate-density lipoprotein, and remnants of very low-density lipoprotein (VLDL) [7, 8]. On the other hand, high-density lipoprotein cholesterol (HDL-c), which is composed of the smallest and densest lipoprotein particles, prevents atherosclerosis [9]. Therefore, the ratio of non-HDL-c to HDL-c (NHHR) serves as a new and comprehensive index for lipid evaluation to cover the array of lipid particles that either promote or inhibit atherosclerosis [10]. Scholarly research has revealed the superior predictive power of the NHHR over conventional lipid metrics for assessing the risk of atherosclerosis and cardiovascular disorders [11, 12]. Additionally, emerging research has revealed the possibility of using the NHHR as an independent predictive marker for conditions such as diabetes and metabolic syndrome, indicating its invaluable contribution to the assessment of metabolic anomalies [13–15].

Despite epidemiological evidence indicating a close correlation between dyslipidaemia marker concentrations and the risk of developing HUA, existing research does not address the potential role of the NHHR in the risk of developing HUA [16, 17]. Using data sourced from the National Health and Nutrition Examination Survey (NHANES), the aim of this study was to uncover the relationship between the NHHR and the risk of developing HUA in U.S. adults. The hypothesis of this study was that there would be a strong correlation between the NHHR and the risk of developing HUA.

## Materials and methods

### Study population

The NHANES is a comprehensive research plan using a complex probability sampling method aimed at the assessment of health and nutritional status among U.S. adult and child populations. Participants in the NHANES engage in health interviews, clinical tests, dietary assessments, and physical examinations [18]. The Ethics Review Board of the National Center for Health Statistics approved the research protocol. More detailed information can be found in the NHANES database. This research ultimately involved 30,937 eligible participants drawn from a pool of 59,842 individuals by merging the NHANES cycles spanning 2007 to 2018. All study participants were aged 20 years or older, were not pregnant, and had complete NHHR and serum uric acid (SUA) data.

### Exposure and outcome definitions

In this study, the NHHR, which is the ratio of non-HDL-c to HDL-c, was used as the exposure variable. Using blood samples obtained from fasting individuals, non-HDL-c is determined by deducting HDL-c from total cholesterol (TC) [19, 20]. HUA was defined as SUA levels ≥ 7 mg/dL in men and ≥ 6 mg/dL in women [21].

### Covariate definitions

The study considered various potential covariates, including age (years), gender, race, income, education, smoking status, diabetes mellitus status, hypertension status, blood pressure, body mass index (BMI, kg/m^2^), waist circumference (WC, cm), and biochemical markers such as glycohemoglobin (HbA1c, %), alanine aminotransferase (ALT, U/L) concentrations, aspartate aminotransferase (AST, U/L) concentrations, gamma-glutamyl transferase (GGT, U/L) concentrations, triglyceride (TG, mmol/L) concentrations, LDL-c concentrations, serum creatinine (SCr, µmol/L) concentrations, and the estimated glomerular filtration rate (eGFR, mL/min/1.73 m^2^). The BMI was categorized as < 25 (normal), 25-29.9 (overweight), or ≥ 30 kg/m^2^ (obese). The eGFR calculation was based on the Chronic Kidney Disease Epidemiology Collaboration (CKD-EPI) formula [22]. Smoking history included both former and current smoking status. Diabetes and hypertension status were indicated by a self-reported history of either condition.

### Statistical analysis

Statistical analyses in this research were conducted using Empower software (http://www.empowerstats.com) and R software (http://www.R-project.org) in compliance with the standards prescribed by the Centers for Disease Control and Prevention (CDC). This research involved the application of a complex, tiered cluster sampling method, incorporating sample weights. Continuous data are presented as the means, while categorical data are expressed as percentages. The weighted Student’s t test and chi-squared test were applied for comparisons across various groups. Logistic and linear regression models were applied to examine the associations of the levels of non-HDL-c and HDL-c and the NHHR with the risk of developing HUA, as well as between those factors and the SUA concentration. The variance inflation factor was further applied to detect multicollinearity in regression analyses. Decision curve analysis (DCA) and receiver operating characteristic (ROC) curve analysis were applied to measure the efficacy of using non-HDL-c, HDL-c, and the NHHR to determine the risk of developing HUA. Subgroup analyses were also performed. Finally, restricted cubic spline (RCS) logistic regression with four knots was applied to investigate the nonlinear associations between the NHHR and the risk of developing HUA, using the median values of the NHHR as references (OR = 1). For observed nonlinear relationships, a two-piecewise linear regression model was applied to define intervals and identify threshold effects. A ***P*** value (two-sided) < 0.05 was considered to indicate statistical significance.

## Results

### Baseline characteristics

A total of 30,937 participants, with an average age of 47.78 years, 48.79% of whom were male, were included in this study (Table 1). Compared with the non-HUA group, the HUA group was older and had more males, individuals with an annual income under $20,000, smokers, and individuals with hypertension and diabetes (*P* < 0.05). Additionally, individuals in this group exhibited increased BMI, WC, systolic blood pressure (SBP), diastolic blood pressure (DBP), HbA1c concentrations, ALT concentrations, AST concentrations, GGT concentrations, TG concentrations, TC concentrations, LDL-c concentrations, non-HDL-c concentrations, SCr concentrations, and SUA concentrations (*P* < 0.01). Conversely, a greater proportion of individuals with education levels above high school, as well as a reduced eGFR and HDL-c concentration, were observed (*P* < 0.001). Additionally, differences in race distribution were also observed (*P* < 0.001). The HUA group exhibited notably greater NHHR values than did the non-HUA group (*P* < 0.001).

**Table 1:** Baseline characteristics of the study population in the NHANES from 2007 to 2018, weighted

	Overall (*N* = 30,937)	Non-HUA group (*N* = 25,570)	HUA group (*N* = 5,367)	*P* value
Age (years)	47.78 ± 0.23	47.17 ± 0.24	50.90 ± 0.32	< 0.001
Male gender, % (SE)	48.79 (0.30)	45.07 (0.33)	67.88 (0.83)	< 0.001
Race, % (SE)				< 0.001
Mexican American	8.62 (0.76)	9.01 (0.78)	6.66 (0.72)	
Non-Hispanic Black	10.66 (0.70)	10.29 (0.67)	12.56 (0.96)	
Non-Hispanic White	66.74 (1.40)	66.45 (1.42)	68.22 (1.52)	
Other Hispanic	5.89 (0.49)	6.17 (0.52)	4.47 (0.43)	
Other Races	8.09 (0.45)	8.08 (0.46)	8.10 (0.57)	
Annual household income (under $20,000), % (SE)	14.21 (0.52)	13.96 (0.55)	15.51 (0.71)	0.015
Education level (above high school), % (SE)	61.11 (0.91)	61.69 (0.93)	58.19 (1.24)	< 0.001
Smokers, % (SE)	44.42 (0.58)	43.54 (0.65)	48.91 (0.93)	< 0.001
Diabetes, % (SE)	9.93 (0.26)	8.89 (0.26)	15.32 (0.70)	< 0.001
Hypertension, % (SE)	32.30 (0.52)	28.69 (0.52)	50.88 (1.02)	< 0.001
SBP (mmHg)	121.96 ± 0.19	121.12 ± 0.20	126.42 ± 0.35	< 0.001
DBP (mmHg)	70.56 ± 0.21	70.34 ± 0.21	71.68 ± 0.29	< 0.001
BMI (kg/m2)	29.08 ± 0.08	28.42 ± 0.08	32.46 ± 0.16	< 0.001
WC (cm)	99.34 ± 0.22	97.52 ± 0.23	108.86 ± 0.39	< 0.001
HbA1c (%)	5.65 ± 0.01	5.62 ± 0.01	5.78 ± 0.02	< 0.001
ALT (U/L)	25.20 ± 0.14	24.01 ± 0.12	31.30 ± 0.49	< 0.001
AST (U/L)	25.22 ± 0.11	24.54 ± 0.12	28.74 ± 0.31	< 0.001
GGT (U/L)	28.14 ± 0.26	25.91 ± 0.27	39.58 ± 0.80	< 0.001
TG (mmol/L)	1.40 ± 0.02	1.32 ± 0.02	1.78 ± 0.04	< 0.001
TC (mmol/L)	4.99 ± 0.01	4.97 ± 0.01	5.10 ± 0.02	< 0.001
LDL-c (mmol/L)	2.94 ± 0.01	2.92 ± 0.01	3.00 ± 0.03	0.007
HDL-c (mmol/L)	1.38 ± 0.01	1.41 ± 0.01	1.24 ± 0.01	< 0.001
Non-HDL-c (mmol/L)	3.61 ± 0.01	3.56 ± 0.01	3.86 ± 0.02	< 0.001
SCr (µmol/L)	78.37 ± 0.26	75.59 ± 0.24	92.65 ± 0.59	< 0.001
eGFR (mL/min/1.73 m2)	94.43 ± 0.31	96.48 ± 0.31	83.89 ± 0.47	< 0.001
SUA (mg/dL)	5.43 ± 0.01	5.00 ± 0.01	7.67 ± 0.02	< 0.001
NHHR	2.92 ± 0.02	2.82 ± 0.02	3.43 ± 0.03	< 0.001

Note: Values for categorical variables are given as weighted percentage (standard error); for continuous variables, as weighted mean ± standard error. Weighted Student’s t-test and chi-squared test were used

Abbreviations: HUA, hyperuricemia; SBP, systolic blood pressure; DBP, diastolic blood pressure; BMI, body mass index; WC, waist circumference; HbA1c, glycohemoglobin; ALT, alanine transaminase; AST, aspartate transaminase; GGT, gamma-glutamyl transferase; TG, triglyceride; TC, total cholesterol; LDL-c, low-density lipoprotein cholesterol; HDL-c, high-density lipoprotein cholesterol; non-HDL-c, high-density lipoprotein cholesterol; Scr, serum creatinine; eGFR, estimated glomerular filtration rate; SUA, serum uric acid; NHHR, non-high-density lipoprotein cholesterol to high-density lipoprotein cholesterol ratio

### Baseline characteristics based on the quantiles of the NHHR

According to the NHHR, the subjects were categorized into four groups based on quantiles (Table 2). The high-NHHR quantile group exhibited greater proportions of males, individuals with an annual income under $20,000, smokers, and individuals with diabetes and hypertension, as well as variations in racial distribution (*P* < 0.05). Furthermore, BMI, WC, SBP, DBP, ALT concentrations, AST concentrations, GGT concentrations, HbA1c values, TG concentrations, TC concentrations, LDL-c concentrations, non-HDL-c concentrations, and SCr concentrations were obviously increased (*P* < 0.001). In contrast, the proportions of individuals with high education levels, high eGFRs, and high HDL-c levels decreased (*P* < 0.01). Compared to those in the lowest NHHR quartile, individuals in the second and third quartiles were older, and those in the fourth quartile were younger (*P* < 0.05). Notably, a higher NHHR was related to increased levels of SUA and a greater prevalence of HUA (9.01% vs. 13.38% vs. 17.31% vs. 25.79%, *P* < 0.001).

**Table 2:** Baseline characteristics of the study population according to the quartiles of the weighted NHHR

	Quartile 1 (0.21, 1.92)	Quartile 2 (1.92, 2.66)	Quartile 3 (2.66, 3.64)	Quartile 4 (3.64, 26.85)	*P* value
Age (years)	47.35 ± 0.39	48.33 ± 0.33	48.15 ± 0.32	47.28 ± 0.27	0.014
Male gender, % (SE)	33.01 (0.78)	42.30 (0.75)	54.27 (0.72)	66.04 (0.69)	< 0.001
Race, % (SE)					< 0.001
Mexican American	6.12 (0.58)	7.82 (0.73)	9.46 (0.89)	11.18 (1.08)	
Non-Hispanic Black	13.74 (0.94)	11.87 (0.78)	9.62 (0.71)	7.33 (0.56)	
Non-Hispanic White	67.82 (1.39)	66.29 (1.34)	66.74 (1.69)	66.04 (1.66)	
Other Hispanic	4.62 (0.46)	5.63 (0.51)	6.36 (0.61)	7.00 (0.64)	
Other Races	7.70 (0.52)	8.39 (0.56)	7.83 (0.52)	8.45 (0.57)	
Annual household income (under $20,000), % (SE)	13.44 (0.64)	14.29 (0.62)	13.67 (0.63)	15.49 (0.83)	0.021
Education level (above high school), % (SE)	67.20 (1.02)	63.34 (1.15)	59.14 (1.06)	54.59 (1.14)	< 0.001
Smokers, % (SE)	40.85 (0.79)	41.85 (0.87)	44.45 (0.79)	50.68 (0.79)	< 0.001
Diabetes, % (SE)	8.61 (0.39)	9.72 (0.43)	9.95 (0.53)	11.51 (0.48)	< 0.001
Hypertension, % (SE)	27.73 (0.89)	31.73 (0.81)	34.14 (0.80)	35.73 (0.80)	< 0.001
SBP (mmHg)	119.92 ± 0.29	120.87 ± 0.34	122.61 ± 0.26	124.55 ± 0.30	< 0.001
DBP (mmHg)	68.16 ± 0.26	69.51 ± 0.28	71.24 ± 0.27	73.44 ± 0.25	< 0.001
BMI (kg/m2)	26.09 ± 0.10	28.67 ± 0.12	30.22 ± 0.12	31.42 ± 0.11	< 0.001
WC (cm)	90.78 ± 0.25	97.80 ± 0.27	102.51 ± 0.32	106.48 ± 0.28	< 0.001
HbA1c (%)	5.47 ± 0.01	5.59 ± 0.01	5.67 ± 0.01	5.86 ± 0.02	< 0.001
ALT (U/L)	21.43 ± 0.29	22.98 ± 0.21	25.32 ± 0.21	31.21 ± 0.31	< 0.001
AST (U/L)	24.95 ± 0.24	24.23 ± 0.17	24.71 ± 0.18	27.03 ± 0.25	< 0.001
GGT (U/L)	25.27 ± 0.51	24.38 ± 0.41	27.73 ± 0.46	35.32 ± 0.71	< 0.001
TG (mmol/L)	0.81 ± 0.01	1.09 ± 0.01	1.44 ± 0.02	2.45 ± 0.04	< 0.001
TC (mmol/L)	4.44 ± 0.02	4.73 ± 0.02	5.08 ± 0.02	5.74 ± 0.02	< 0.001
LDL-c (mmol/L)	2.25 ± 0.01	2.79 ± 0.02	3.19 ± 0.02	3.73 ± 0.02	< 0.001
HDL-c (mmol/L)	1.82 ± 0.01	1.44 ± 0.01	1.24 ± 0.00	1.00 ± 0.00	< 0.001
Non-HDL-c (mmol/L)	2.62 ± 0.01	3.28 ± 0.01	3.84 ± 0.01	4.73 ± 0.02	< 0.001
SCr (µmol/L)	75.96 ± 0.54	77.03 ± 0.33	79.34 ± 0.46	81.21 ± 0.44	< 0.001
eGFR (mL/min/1.73 m2)	95.52 ± 0.46	94.00 ± 0.44	93.86 ± 0.44	94.30 ± 0.37	0.007
SUA (mg/dL)	4.90 ± 0.02	5.24 ± 0.02	5.60 ± 0.02	6.01 ± 0.02	< 0.001
HUA, % (SE)	9.01 (0.43)	13.38 (0.55)	17.31 (0.54)	25.79 (0.65)	< 0.001

Note: Values for categorical variables are given as weighted percentage (standard error); for continuous variables, as weighted mean ± standard error. Weighted Student’s t-test and chi-squared test were used

### Association between the NHHR and the risk of developing HUA

**Fig. 1:**
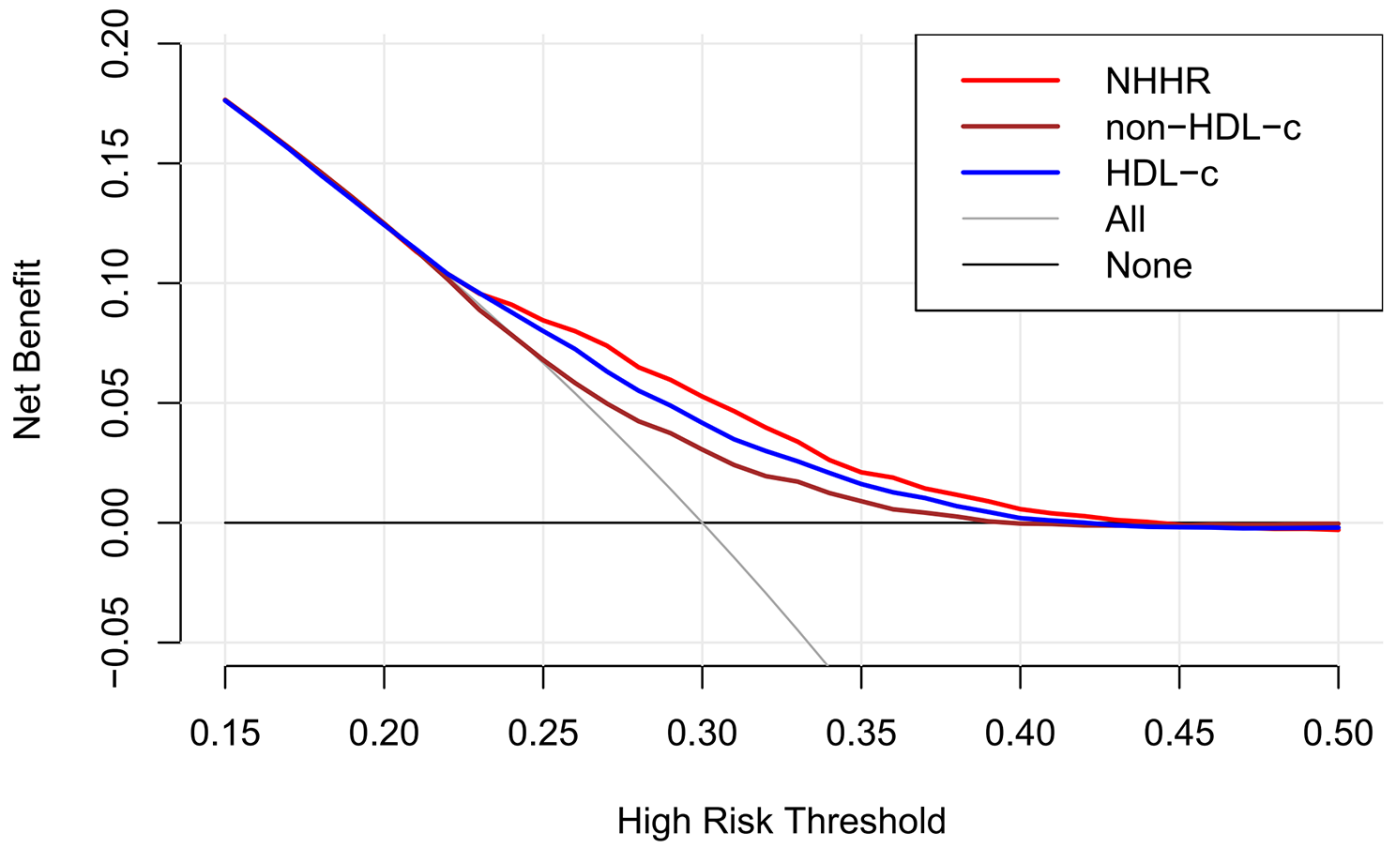
DCA results

**Fig. 2:**
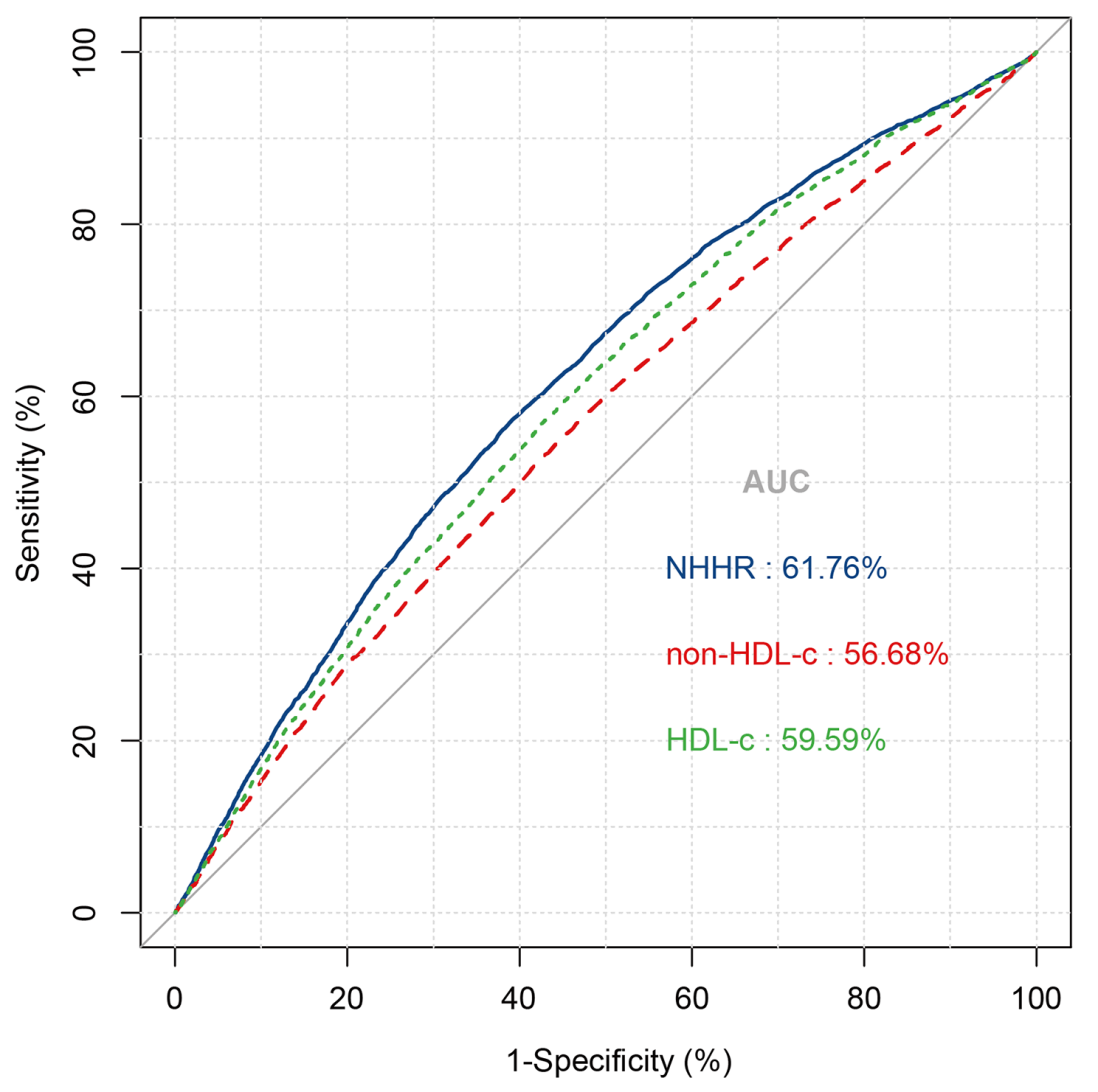
ROC results

The NHHR is positively correlated with the prevalence of HUA, a statistically significant relationship that persists across unadjusted, preliminarily adjusted, and fully adjusted logistic regression models (Table 3). Upon complete adjustment, each unit increase in the NHHR was associated with a 10% increase in the risk of developing HUA (OR = 1.10, 95% CI: 1.05–1.16, *P* < 0.001) (Supplementary Tables 1 and 2). When the NHHR was divided into quartiles, participants in the highest quartile clearly faced a greater risk, with a 0.94-fold increase compared to those in the lowest quartile (OR = 1.94, 95% CI: 1.62–2.33, *P* < 0.001). Using SUA levels as the dependent variable, linear regression analysis revealed a significant correlation between the NHHR and SUA (β = 0.06, 95% CI: 0.04–0.08, *P* < 0.001) (Table 4). Based on the ROC curve results, the areas under the curve (AUCs) for the NHHR, non-HDL-c, and HDL-c were 61.76%, 56.68%, and 59.59%, respectively. Additionally, DCA showed that compared with non-HDL-c and HDL-c concentrations, the NHHR offers a greater net benefit (Figs. 1 and 2).

**Table 3:** Logistic regression analysis results for the association between the NHHR and the risk of developing HUA.

HUA	OR (95%CI) *P* value		
	Model 1	Model 2	Model 3
Continuous			
Non-HDL-c	1.22 (1.19, 1.25) < 0.001	1.24 (1.21, 1.27) < 0.001	1.14 (1.08, 1.21) < 0.001
HDL-c	0.37 (0.34, 0.40) < 0.001	0.39 (0.36, 0.43) < 0.001	0.76 (0.64, 0.89) 0.001
NHHR	1.25 (1.23, 1.27) < 0.001	1.25 (1.22, 1.27) < 0.001	1.10 (1.05, 1.16) < 0.001
Categories			
Quantile 1	reference	reference	reference
Quantile 2	1.43 (1.30, 1.58) < 0.001	1.42 (1.29, 1.57) < 0.001	1.17 (0.99, 1.38) 0.068
Quantile 3	1.95 (1.77, 2.14) < 0.001	1.92 (1.74, 2.11) < 0.001	1.48 (1.25, 1.75) < 0.001
Quantile 4	2.99 (2.74, 3.27) < 0.001	3.00 (2.73, 3.29) < 0.001	1.94 (1.62, 2.33) < 0.001
P for trend	< 0.001	< 0.001	< 0.001

OR: odds ratio

95% CI: 95% confidence interval

Model 2: adjusted for age, gender, and race

Model 3: adjusted for age, gender, and race, annual household income, education level, smokers, diabetes, hypertension, SBP, DBP, BMI, WC, HbA1c, ALT, AST, GGT, TG, SCr, and eGFR.

**Table 4:** Linear regression analysis results for the association between the NHHR and the SUA concentration

SUA	β (95%CI) *P* value		
	Model 1	Model 2	Model 3
Continuous			
Non-HDL-c	0.19 (0.17, 0.20) < 0.001	0.17 (0.16, 0.18) < 0.001	0.08 (0.06, 0.11) < 0.001
HDL-c	-0.84 (-0.87, -0.80) < 0.001	-0.57 (-0.61, -0.53) < 0.001	-0.15 (-0.21, -0.09) < 0.001
NHHR	0.23 (0.22, 0.24) < 0.001	0.17 (0.16, 0.18) < 0.001	0.06 (0.04, 0.08) < 0.001
Categories			
Quantile 1	reference	reference	reference
Quantile 2	0.29 (0.25, 0.34) < 0.001	0.23 (0.19, 0.28) < 0.001	0.07 (0.01, 0.13) 0.013
Quantile 3	0.62 (0.58, 0.67) < 0.001	0.48 (0.44, 0.52) < 0.001	0.23 (0.17, 0.29) < 0.001
Quantile 4	0.99 (0.94, 1.03) < 0.001	0.77 (0.72, 0.81) < 0.001	0.39 (0.32, 0.46) < 0.001
P for trend	< 0.001	< 0.001	< 0.001

95% CI: 95% confidence interval

Model 2: adjusted for age, gender, and race

Model 3: adjusted for age, gender, and race, annual household income, education level, smokers, diabetes, hypertension, SBP, DBP, BMI, WC, HbA1c, ALT, AST, GGT, TG, SCr, and eGFR.

### Subgroup analysis and threshold effect

**Fig. 3:**
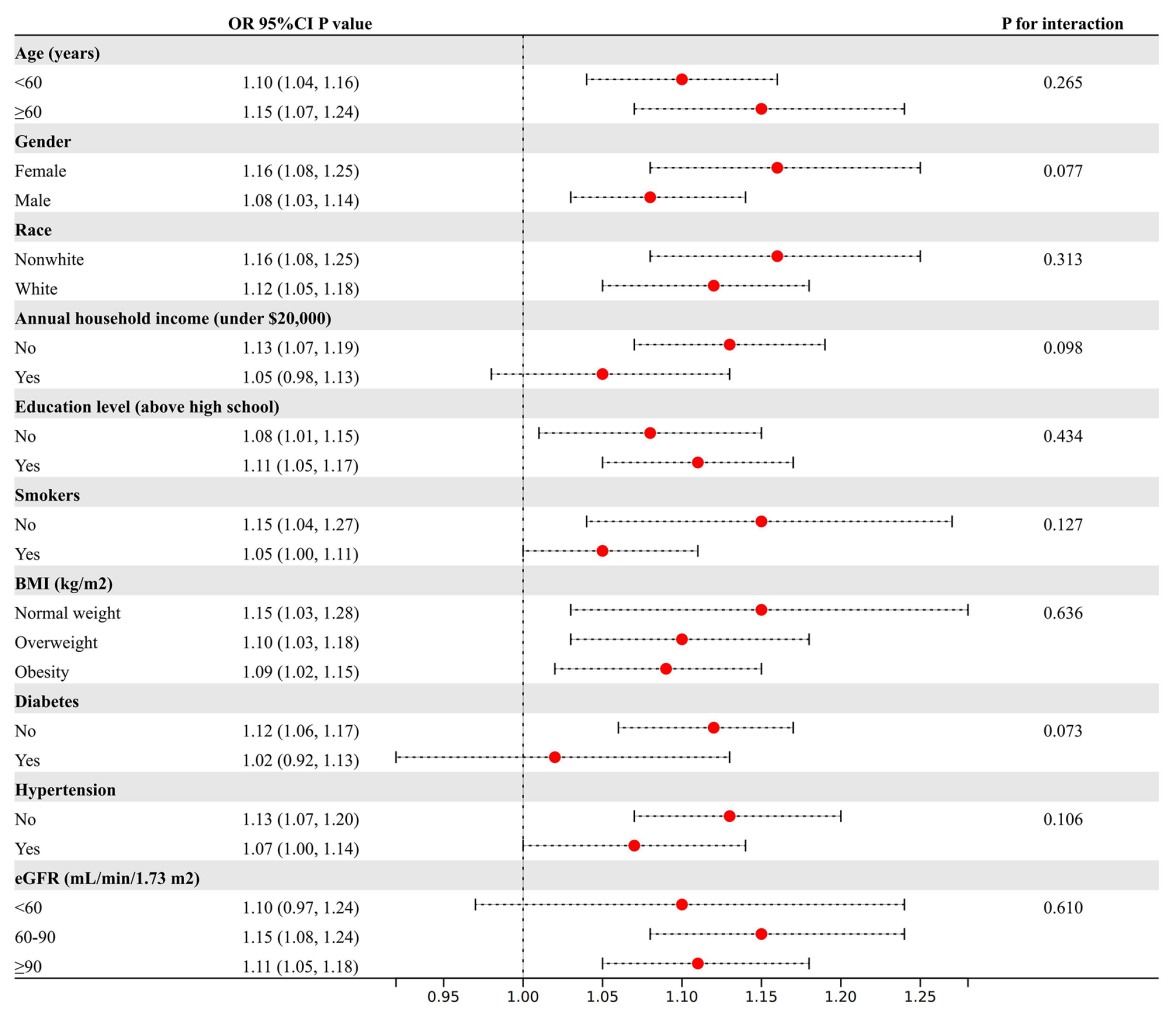
Subgroup analyses

**Fig. 4:**
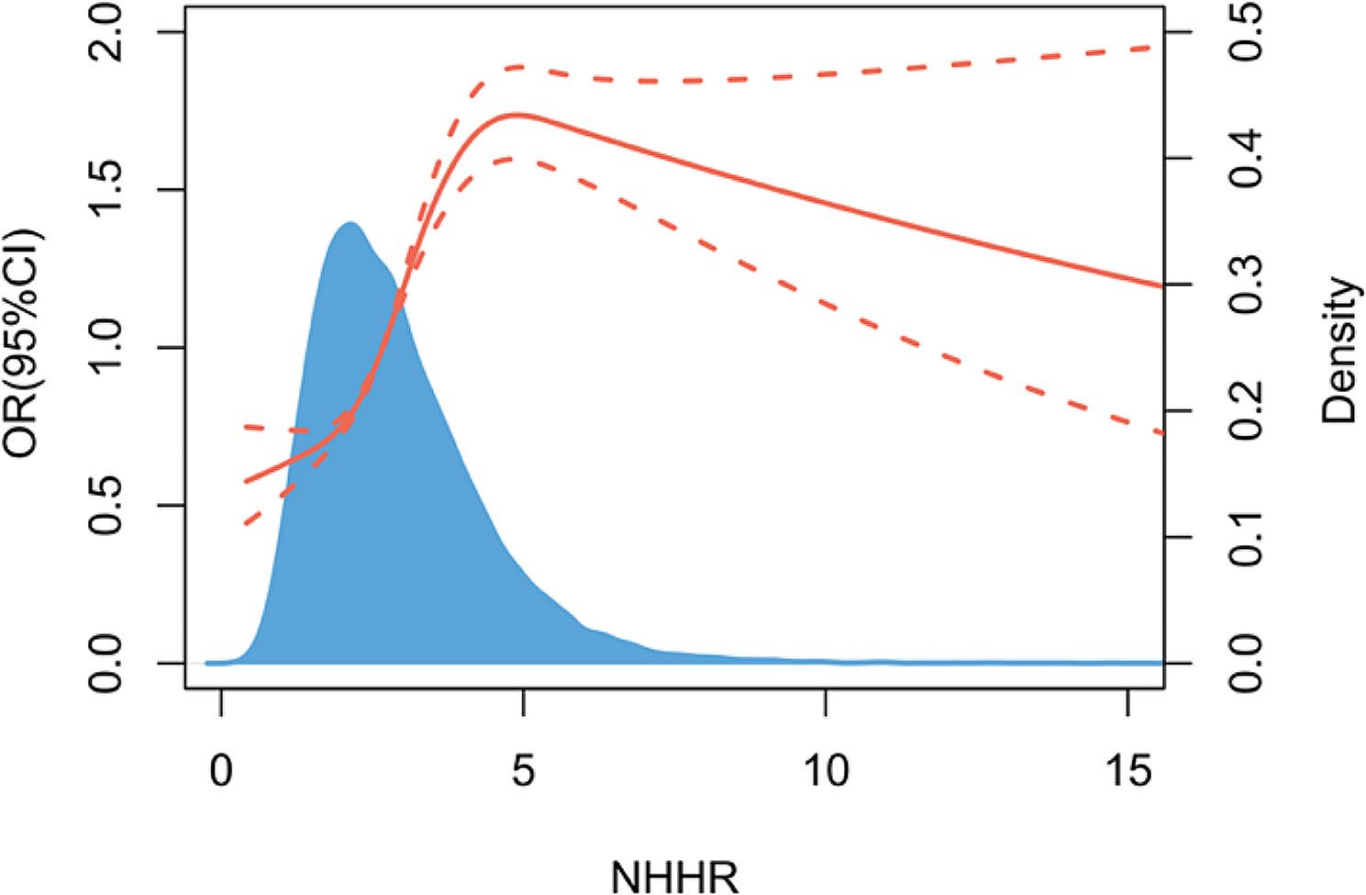
Results of RCS analysis

Subgroup analyses were carried out considering factors such as age, gender, race, annual household income, education level, smoking status, BMI, diabetes status, hypertension status, and the eGFR to evaluate the reliability of this relationship between the NHHR and the risk of developing HUA across different populations (Fig. 3). The results indicated that these factors had no significant impact on the relationship (*P* > 0.05). Interestingly, the RCS results showed a nonlinear, inverted U-shaped relationship between the NHHR and the risk of developing HUA across the entire population (Fig. 4). Further investigation using a two-piecewise linear regression identified a breakpoint at 5.14 (Table 5). To the left of the breakpoint, a positive correlation existed between the NHHR and the risk of developing HUA, as indicated by an OR of 1.25 and a 95% CI ranging from 1.18 to 1.32. To the right of the breakpoint, the NHHR and the risk of developing HUA were inversely correlated, with an OR of 0.77 and a 95% CI of 0.68 to 0.88. There was a significant change across the breakpoint (*P* < 0.001).

**Table 5:** Threshold effect analysis of the association between the NHHR and the risk of developing HUA using a two-piecewise linear regression model

Model	OR (95% CI), *P* value
Fitting by standard linear model	
	1.10 (1.05, 1.16) < 0.001
Fitting by two-piecewise linear model	
Breakpoint (K)	5.14
OR1 (< 5.14)	1.25 (1.18, 1.32) < 0.001
OR2 (> 5.14)	0.77 (0.68, 0.88) < 0.001
OR2/OR1	0.62 (0.54, 0.71) < 0.001
P for logarithmic likelihood ratio	< 0.001

adjusted for age, gender, and race, annual household income, education level, smokers, diabetes, hypertension, SBP, DBP, BMI, WC, HbA1c, ALT, AST, GGT, TG, SCr, and eGFR.

## Discussion

This groundbreaking population-based study explored the connection between the NHHR and the risk of developing HUA. Compared to traditional lipid indicators, a higher NHHR is strongly associated with a greater risk of developing HUA.

Usually, LDL-c is the focus for managing dyslipidaemia [23]. However, non-HDL-c, which includes all plasma lipoproteins except for HDL-c, are recognized as a significant risk factor for cardiovascular diseases [24, 25]. Therefore, non-HDL-c has gradually become a coprimary or primary target in dyslipidaemia management [24]. Several prior studies from China have also established an independent association between the non-HDL-c concentration and the risk of developing HUA [16, 20]. Moreover, some scholars have also reported that a decrease in HDL-c levels is a key risk factor for HUA [26]. Emerging evidence suggests that lipid ratios might be valuable indicators for various diseases, including cardiovascular diseases, diabetes, and metabolic syndrome [13, 27–29]. The NHHR was proposed to encompass all information related to pro-atherosclerotic and anti-atherosclerotic lipid particles, representing the balance between lipoproteins [10, 30]. Studies have demonstrated that the NHHR significantly surpasses traditional lipid parameters in the assessment of atherosclerosis [11]. Similarly, in the field of metabolic disorders, past studies have shown that the NHHR has excellent predictive ability for diabetes, metabolic syndrome, and insulin resistance (IR), surpassing individual lipid indicators such as non-HDL-c, HDL-c, and LDL-c [13, 15]. Nevertheless, there has been limited research on the correlation between lipid ratios and the risk of developing HUA, making it unclear whether the NHHR can serve as a useful marker for HUA. This study revealed that compared with the individual measurements of non-HDL-c and HDL-c, the NHHR has superior diagnostic predictive value for the risk of developing HUA. Additionally, further RCS analysis indicated a nonlinear, inverse U-shaped association between the NHHR and the risk of developing HUA, consistent with previous studies on the connection between the NHHR and suicidal ideation [31]. This study extends the use of lipid ratios and fills the gaps in previous research, suggesting that the NHHR might be a promising marker for predicting HUA. Notably, cohort research has confirmed a close relationship between dyslipidaemia and the incidence of HUA [32–34]. Hence, from the perspective of lipid management, the prevention and treatment of HUA could offer important clinical benefits. Current research has also attempted to focus on non-HDL-c as a key point in lipid management [24]. Whether NHHR is a novel target for lipid management requires further investigation. Finally, subgroup analyses and interaction tests did not identify specific populations; however, further analysis is warranted for other groups, such as older nonwhite women.

Several plausible factors, such as IR, oxidative stress, inflammation, lifestyle, and the use of lipid-lowering medications, might contribute to revealing the association between dyslipidaemia marker concentrations and the risk of developing HUA. IR, a common factor underlying both conditions, impairs the body’s ability to use insulin efficiently, leading to disrupted lipid metabolism and increased uric acid production [35, 36]. Additionally, an imbalance between free radicals and antioxidants causes oxidative stress, resulting in lipid peroxidation and uric acid accumulation [37, 38]. Chronic inflammation, often observed in individuals with dyslipidaemia, can also stimulate uric acid synthesis and impair its excretion [39]. Researchers have also discovered that patients with hyperlipidaemia and HUA share similar dietary behaviours, such as consuming large amounts of alcohol and fatty foods [40]. Interestingly, the administration of lipid-lowering medications is associated with alterations in SUA levels [41].

## Strengths and limitations of the study

This study has several strengths. It was based on data from the National Health and Nutrition Examination Survey (NHANES) database, which is nationally representative and has a relatively large sample size. This study included effective control for potential confounding factors, enhancing the reliability of the results. RCS analysis was also employed to further investigate nonlinear relationships and assess the reliability of the results across different populations through subgroup analysis.

However, there are also some limitations to the current research. First, the use of a cross-sectional design prevents us from inferring causality; therefore, prospective cohort studies and intervention trials are essential to elucidate the causality of these associations. Second, this study did not account for certain potential confounders, including the use of uric acid-lowering and lipid-lowering medications or dietary patterns marked by high consumption of alcohol and fatty foods, which could skew the results. Furthermore, as the sample comes from the US population, the applicability of the results to other populations needs additional verification.

## Conclusion

In a nationally representative study conducted among adults aged ≥ 20 years, the NHHR was associated with the risk of developing HUA. Implementing lipid management to improve the NHHR could help in assessing, preventing, and treating HUA.

### Electronic supplementary material

Below is the link to the electronic supplementary material.

Supplementary Material 1

Supplementary Material 2

Supplementary Material 3

## Data Availability

The data are sourced from the NHANES database, which is a publicly accessible and free resource (https://www.cdc.gov/nchs/nhanes).
