# Supplementary material for: The relationship between non-high-density lipoprotein cholesterol to high-density lipoprotein cholesterol ratio (NHHR) and hyperuricaemia
Source: Lipids Health Dis. 2024 Jun 21;23:187. doi: 10.1186/s12944-024-02171-4 (PMC11191326; doi:10.1186/s12944-024-02171-4)
Supplement: Supplementary file 1 — Supplementary Material 1 [file 12944_2024_2171_MOESM1_ESM.docx]

| **Supplementary Table 1** Multivariate logistic regression models of HUA. | | | | |
| --- | --- | --- | --- | --- |
|  | OR | 95%CI lower | 95%CI upper | P value |
| NHHR | 1.104 | 1.052 | 1.158 | <0.001 |
| Age (years) | 1.017 | 1.015 | 1.019 | <0.001 |
| Male (vs. female) | 2.380 | 2.083 | 2.718 | <0.001 |
| Race (vs. Mexican American) |  |  |  |  |
| Non-Hispanic Black | 2.053 | 1.683 | 2.504 | <0.001 |
| Non-Hispanic White | 1.127 | 0.941 | 1.350 | 0.195 |
| Other Hispanic | 1.089 | 0.864 | 1.373 | 0.471 |
| Other Races | 2.196 | 1.758 | 2.742 | <0.001 |
| Under $20,000 (vs. no) | 1.016 | 0.890 | 1.159 | 0.818 |
| Above high school (vs. no) | 1.011 | 0.904 | 1.131 | 0.848 |
| Smokers (vs. no) | 1.016 | 0.912 | 1.133 | 0.772 |
| Diabetes (vs. no) | 1.040 | 0.867 | 1.247 | 0.674 |
| Hypertension (vs. no) | 1.555 | 1.377 | 1.756 | <0.001 |
| SBP (mmHg) | 1.007 | 1.003 | 1.010 | <0.001 |
| DBP (mmHg) | 1.005 | 1.003 | 1.008 | <0.001 |
| BMI (kg/m2) | 1.032 | 1.010 | 1.054 | 0.003 |
| WC (cm) | 1.023 | 1.013 | 1.032 | <0.001 |
| HbA1c (%) | 1.136 | 1.110 | 1.164 | <0.001 |
| ALT (U/L) | 1.008 | 1.004 | 1.012 | <0.001 |
| AST (U/L) | 0.998 | 0.994 | 1.001 | 0.159 |
| GGT (U/L) | 1.003 | 1.002 | 1.005 | <0.001 |
| TG (mmol/L) | 1.132 | 1.073 | 1.195 | <0.001 |
| SCr (μmol/L) | 1.017 | 1.016 | 1.018 | <0.001 |
| eGFR (mL/min/1.73 m2) | 0.955 | 0.951 | 0.960 | <0.001 |

| **Supplementary Table 2** Multivariate linear regression models of SUA concentration. | | | | |
| --- | --- | --- | --- | --- |
|  | β | 95%CI lower | 95%CI upper | P value |
| NHHR | 0.063 | 0.043 | 0.082 | <0.001 |
| Age (years) | 0.011 | 0.010 | 0.012 | <0.001 |
| Male (vs. female) | 1.045 | 0.996 | 1.094 | <0.001 |
| Race (vs. Mexican American) |  |  |  |  |
| Non-Hispanic Black | 0.340 | 0.268 | 0.413 | <0.001 |
| Non-Hispanic White | 0.005 | -0.059 | 0.069 | 0.871 |
| Other Hispanic | -0.030 | -0.111 | 0.050 | 0.462 |
| Other Races | 0.392 | 0.311 | 0.473 | <0.001 |
| Under $20,000 (vs. no) | 0.009 | -0.042 | 0.060 | 0.731 |
| Above high school (vs. no) | -0.012 | -0.055 | 0.031 | 0.589 |
| Smokers (vs. no) | -0.020 | -0.062 | 0.022 | 0.354 |
| Diabetes (vs. no) | 0.019 | -0.058 | 0.096 | 0.626 |
| Hypertension (vs. no) | 0.205 | 0.156 | 0.255 | <0.001 |
| SBP (mmHg) | 0.003 | 0.001 | 0.004 | <0.001 |
| DBP (mmHg) | 0.008 | 0.006 | 0.009 | <0.001 |
| BMI (kg/m2) | 0.021 | 0.012 | 0.029 | <0.001 |
| WC (cm) | 0.015 | 0.012 | 0.019 | <0.001 |
| HbA1c (%) | 0.065 | 0.050 | 0.080 | <0.001 |
| ALT (U/L) | 0.004 | 0.002 | 0.006 | <0.001 |
| AST (U/L) | -0.001 | -0.003 | 0.000 | 0.133 |
| GGT (U/L) | 0.002 | 0.002 | 0.003 | <0.001 |
| TG (mmol/L) | 0.059 | 0.037 | 0.081 | <0.001 |
| SCr (μmol/L) | 0.010 | 0.009 | 0.012 | <0.001 |
| eGFR (mL/min/1.73 m2) | -0.028 | -0.029 | -0.026 | <0.001 |

| Variance inflation factor (VIF) | |
| --- | --- |
| Variables | VIF |
| NHHR | 1.9 |
| Age (years) | 3.4 |
| Gender | 1.5 |
| Race | 1.1 |
| Annual household income | 1.1 |
| Education level | 1.1 |
| Smokers | 1.1 |
| Diabetes | 1.7 |
| Hypertension | 1.4 |
| SBP (mmHg) | 1.6 |
| DBP (mmHg) | 1.3 |
| BMI (kg/m2) | 7.6 |
| WC (cm) | 8.1 |
| HbA1c (%) | 1.7 |
| ALT (U/L) | 2.8 |
| AST (U/L) | 2.5 |
| GGT (U/L) | 1.3 |
| TG (mmol/L) | 1.8 |
| Scr (μmol/L) | 2.0 |
| eGFR (ml/min/1.73 m2) | 3.8 |
