# Supplementary material for: The relationship between non-high-density lipoprotein cholesterol to high-density lipoprotein cholesterol ratio (NHHR) and hyperuricaemia
Source: Lipids Health Dis. 2024 Jun 21;23:187. doi: 10.1186/s12944-024-02171-4 (PMC11191326; doi:10.1186/s12944-024-02171-4)
Supplement: Supplementary file 2 — Supplementary Material 2 [file 12944_2024_2171_MOESM2_ESM.pdf]

SPRINGER NATURE

Author Services

Editing Certificate

This document certifies that the manuscript

The relationship between non-high-density lipoprotein cholesterol to high-density lipoprotein cholesterol ratio (NHHR) and hyperuricaemia

prepared by the authors

Zhaoxiang Wang, Menghuan Wu, Ruiqin Du, Fengyan Tang, Mengjiao Xu, Tian Gu, Qichao Yang

was edited for proper English language, grammar, punctuation, spelling, and overall style by one or more of the highly qualified native English speaking editors at SNAS.

This certificate was issued on **May 29, 2024** and may be verified on the SNAS website using the verification code **7976-457E-7413-111B-ADE2** .

Neither the research content nor the authors' intentions were altered in any way during the editing process. Documents receiving this certification should be English-ready for publication; however, the author has the ability to accept or reject our suggestions and changes. To verify the final SNAS edited version, please visit our verification page at secure.authorservices.springernature.com/certificate/verify.
If you have any questions or concerns about this edited document, please contact SNAS at support@as.springernature.com.

SNAS provides a range of editing, translation, and manuscript services for researchers and publishers around the world.
For more information about our company, services, and partner discounts, please visit authorservices.springernature.com.
