# Supplementary material for: The relationship between non-high-density lipoprotein cholesterol to high-density lipoprotein cholesterol ratio (NHHR) and hyperuricaemia
Source: Lipids Health Dis. 2024 Jun 21;23:187. doi: 10.1186/s12944-024-02171-4 (PMC11191326; doi:10.1186/s12944-024-02171-4)
Supplement: Supplementary file 3 — Supplementary Material 3 [file 12944_2024_2171_MOESM3_ESM.pdf]

CA4-00237.docx

by 158 158

Submission date: 09-Apr-2024 02:44PM (UTC+0300)
Submission ID: 2344495091
File name: CA4-00237.docx (408.87K)
Word count: 3985
Character count: 24377

The relationship between non-high-density lipoprotein cholesterol to high-density lipoprotein cholesterol ratio (NHHR) and hyperuricemia

Zhaoxiang Wang[1,&], Menghuan Wu[2,&], Ruiqin Du[3], Fengyan Tang[1], Mengjiao Xu[4], Tian Gu[4], Qichao Yang[4,*]

[1] Department of Endocrinology, Affiliated Kunshan Hospital of Jiangsu University, Kunshan, Jiangsu, 215300, China

[2] Department of Cardiology, Xuyi People's Hospital, Xuyi, Jiangsu, 211700, China

[3] Department of Endocrinology, PLA Rocket Force Characteristic Medical Center, Beijing, 100088, China

[4] Department of Endocrinology, Affiliated Wujin Hospital of Jiangsu University, Changzhou, Jiangsu, 213017, China; Wujin Clinical College of Xuzhou Medical University, Changzhou, Jiangsu, 213017, China

& **These authors contributed equally to this work.**

*** Correspondence**:

Qichao Yang, yangqichao@wjrmyy.cn

ABSTRACT

Purpose: The ratio of non-high-density lipoprotein cholesterol (non-HDL-c) to HDL-c (NHHR) is a novel comprehensive lipid index. The purpose of this study is to examine the association between NHHR and the prevalence of hyperuricemia in the U.S. adult population.

Methods: This cross-sectional study collect data from the 2007 to 2018 cycles of the National Health and Nutrition Examination Survey (NHANES). Hyperuricemia was defined as serum uric acid (SUA) levels ≥7 mg/dL in men and ≥ 6 mg/dL in women. Multivariable regression models and restricted cubic spline (RCS) analyses were employed to analysis the relation in NHHR and hyperuricemia. Subgroup analyses and interaction tests were also performed.

Results: The prevalence of hyperuricemia increases with the levels of NHHR (9.01% vs. 13.38% vs.17.31% vs. 25.79%, ***P***<0.001). After adjusted, NHHR is independently associated with hyperuricemia (OR=1.10, ***P***<0.001). Moreover, than the lowest group of NHHR, the risk of developing hyperuricemia significantly increases in the fourth quartile group (OR=1.94, 95%CI:1.62-2.33, ***P***<0.001). This association was consistent across subgroups. Through restricted cubic spline (RCS) analysis, an inverted U-shaped relation existed in NHHR and hyperuricemia risk.

Conclusions: NHHR was found to be closely linked to an increased risk of hyperuricemia. Further studies on NHHR could be beneficial in the prevention and therapy of hyperuricemia.

Keywords: Hyperuricemia; NHHR; Nonlinear relationship; NHANES

1. Introduction

Hyperuricemia is a typical metabolic disease with feature of an elevated level of uric acid in the plasma, exceeding the standard value. Not only does hyperuricemia serve as an early stage and primary cause of gout, but it also poses a important risk factor for cardiovascular diseases, DM, obesity, and increased mortality rates [1-4]. Nowadays, the global occurrence of it is increasing, imposed a considerable burden worldwide [5-6]. Despite this, its treatment continues to be less than optimal.

The non-HDL-c encompasses all potentially atherogenic cholesterol found in various lipoprotein particles, such as LDL-c, lipoprotein (a), remnants of very low-density lipoprotein [7-8]. High-density lipoprotein cholesterol (HDL-c), prevents atherosclerosis [9]. Thus, as a novel and comprehensive lipid index, the NHHR index was proposed to cover the array of lipid particles that either promote or inhibit atherosclerosis [10]. Scholarly research has cast light on the superior predictive power of NHHR over conventional lipid metrics in assessing the risk of atherosclerosis and cardiovascular disorders [11-12]. Additionally, emerging research have also unveiled NHHR's capability to serve as an independent predictive marker for conditions such as diabetes and metabolic syndrome, indicating its invaluable contribution to the assessment of metabolic anomalies [13-15].

Despite epidemiological evidence indicating a close correlation between dyslipidemia and hyperuricemia, existing research does not address the potential role of NHHR in hyperuricemia [16-17]. Drawing on the NHANES database, this study aims to uncover the relationship between NHHR and hyperuricemia in U.S. adults. It was hypothesized that a relationship exists between NHHR and the risk of hyperuricemia.

2. Materials and methods

2.1 Data source

NHANES is a comprehensive research program using a complex, probability sampling method, aimed at the assessment of health and nutritional status among the USA adult and child populations. Participants in NHANES engage in health interviews, clinical tests, dietary assessments, and physical examinations [18]. The NCHS Ethics Review Board approved the research protocol. More detailed information could be accessed at NHANES database. This research ultimately incorporated 30,937 eligible participants, drawn from a pool of 59,842 individuals by merging the NHANES cycles spanning 2007 to 2018. All study participants were

aged 20 years or older, were not pregnant, and had complete NHHR and serum uric acid (SUA) data.

2.2 Exposure and outcome definitions

NHHR served as the exposure variable in the study. In fasting individuals, non-HDL-c is obtained by deducting HDL-c from TC in their lipid profiles [19-20]. On other hand, hyperuricemia is defined by SUA levels of 7 mg/dL [21].

2.3 Covariate definitions

This study included demographics (age, gender, and race), income, education levels, DM, hypertension, SBP, BMI, WC, and levels of glycohemoglobin (HbA1c, %), alanine aminotransferase (ALT, U/L), aspartate aminotransferase (AST, U/L), GGT, TG, LDL-c, eGFR as potential covariates. BMI was categorized as <25 (normal), 25-29.9 (overweight), ≥ 30 kg/m^2 (obese) for participants. The eGFR calculation was based on the CKD-EPI formula, considering age, gender, Scr [22]. Smoking history included both former and present smoking status. Diabetes and hypertension were assessed based on a self-reported history. Detailed measures of all these factors in present research were available in the NHANES database .

2.4 Statistical analysis

The Empower and R software were applied to statistical treat the data, strictly adhering to the guidelines of the CDC. A complex cluster survey method was employed, incorporating sample weights . Continuous variables were presented as means with SD, while categorical variables were expressed as ratio. The weighted t-test and chi-squared test were employed to compare continuous and categorical variables across various groups. Logistic regression models were applied to examine the relationship among the levels of non-HDL-c, NHHR, and hyperuricemia, as well as between these factors and SUA, respectively. The VIF was applied to evaluate

the level of collinearity in different factors. Decision curve analysis (DCA) and ROC were used to measure the performance of non-HDL-c, NHHR on hyperuricemia. Subgroup analyses were also performed. Finally, a RCS logistic analysis with four knots to clarify the nonlinear associations of NHHR and hyperuricemia, using the median values of NHHR as references (OR=1). For observed non-linear relationships, linear regression analysis was applied to define intervals and identify threshold effects. A two-side ***P*** value <0.05 was regarded as significant judgement criteria.

3. Results

3.1 Baseline data of participants

A total of 30,937 participants were included in this research, with mean age of 47.78 years, including 48.79% males and 51.21% females (**Table 1**). According to the comparison result in different group, the hyperuricemia group was older and showed a higher ratio of males, individuals with an annual income under $20,000, smokers, and those with hypertension and diabetes (***P***<0.05). Additionally, levels of SBP, DBP, BMI, WC, AST, TC, LDL-c, non-HDL-c, Scr, were higher in this group (***P***<0.01). But the proportion of cases with higher education level, alongside reductions in eGFR and HDL-c levels (***P***<0.001). Differences in race distribution between the two groups were also observed (***P***<0.001). Notably, the NHHR level was significantly higher in the hyperuricemia group than the non-hyperuricemia group (***P***<0.001).

3.2 Baseline data based on the quantiles of NHHR levels.

Research objects were divided into four groups based on quantiles of the NHHR levels (**Table 2**). According to the comparison result , the high NHHR quantile group exhibited higher proportions of males, individuals with an annual income under $20,000, smokers, and those with diabetes and hypertension, along with differences in race (***P***<0.05). Furthermore, levels of SBP, DBP, BMI, WC, ALT, HbA1c, TG, TC, non-HDL-c, were obviously increased (***P***<0.001). In contrary, the ratio of individuals

with higher education level and reductions in eGFR and HDL-c levels (***P***<0.01). It was observed that, than the lowest NHHR quartile, the second and third quartiles were older, and the fourth quartile was younger (***P***<0.05). Notably, an increase in NHHR levels was related to higher SUA levels and a higher prevalence of hyperuricemia (9.01% vs. 13.38% vs.17.31% vs. 25.79%, ***P***<0.001).

3.3 Association between NHHR and hyperuricemia

NHHR levels is positive related to the occurrence of hyperuricemia, a relationship that is statistically significant and remains stable across unadjusted, preliminarily adjusted, and fully adjusted logistic regression models (**Table 3**). Upon complete adjustment, each unit increase in NHHR is associated with a 10% increase in the risk of developing hyperuricemia (OR=1.10, ***P***<0.001) (**Attachment 1**). When NHHR is categorized into quartiles, participants in the highest quartile of NHHR face a obvious higher risk, with a 0.94-fold increase in hyperuricemia risk than the lowest quartile (OR=1.94, ***P***<0.001). In conducting a linear regression method with SUA levels, a close correlation was also discovered between NHHR and SUA concentration (***P***<0.001) (**Table 4**). According to the ROC result, the AUC for NHHR, non-HDL-c, and HDL-c are 61.76%, 56.68%, and 59.59%, respectively. Additionally, DCA analysis demonstrates a higher net benefit of NHHR compared to HDL-c (**Figure 1-2**).

Figure 1 DCA results

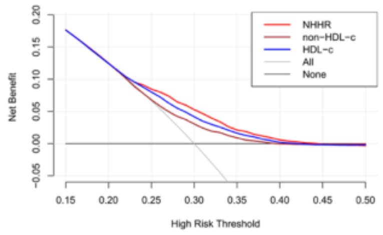

Figure 2 ROC results

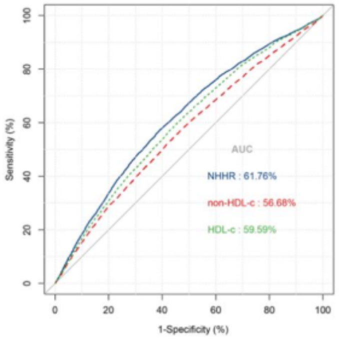

3.4 Subgroup analysis and threshold effect

Subgroup analyses were conducted based on age,race, annual household income, BMI, DM, eGFR levels to evaluate the stability of the relationship in NHHR and hyperuricemia across different populations (**Figure 3**). The results indicated that these variables had no obvious influence on that relationship (***P*** > 0.05). Interestingly, RCS result indicated an inverted U-shaped nonlinear relationship across the entire population (**Figure 4**). Further investigation using a two-piecewise linear method identified a breakpoint (K) at 5.14. To the left of the breakpoint, there exist positive association, with an OR of 1.25, 95% CI of 1.18 to 1.32. To the right of the breakpoint, there exist inverse association, with an OR of 0.77, 95% CI of 0.68 to 0.88. There was a significant change across the breakpoint (***P*** <0.001).

Figure 3 Subgroup analyses

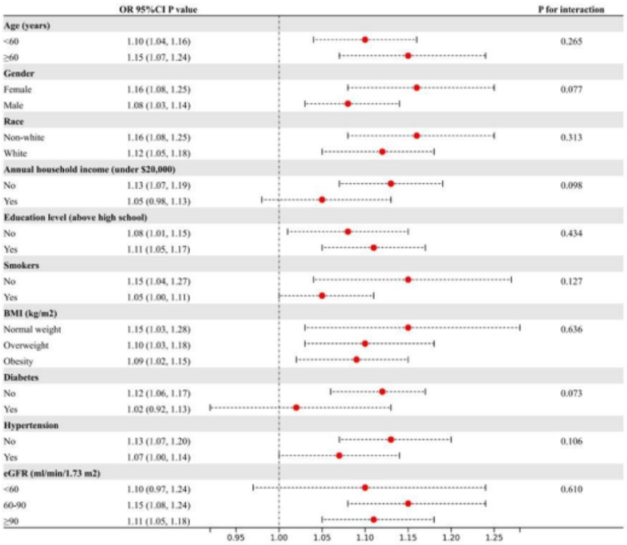

Figure 4 The RCS result. (Adjusted for age, gender, race, DM, SBP, DBP, BMI, WC, GGT, TG, Scr)

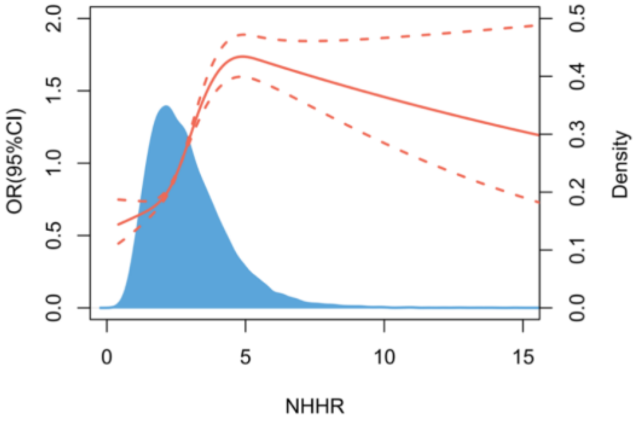

4. Discussion

This is the first population-based research to investigate the relationship in NHHR and hyperuricemia risk. Compared to traditional lipid indicators, higher NHHR level is closely related to increased risk of hyperuricemia.

Usually, LDL-c is the primary target in the management of dyslipidemia [23]. However, non-HDL-c, which includes all plasma lipoproteins except for HDL-c, is considered an independent risk factor or predictor for cardiovascular diseases [24-25]. It has gradually become a co-primary or primary goal in dyslipidemia management [24]. Several prior studies from China have also established an independent link between non-HDL-c and hyperuricemia [16,20]. Moreover, some scholars have also reported that a decrease in HDL-c levels is a key risk factor for hyperuricemia [26]. Emerging evidence suggests lipid ratios might be more valuable indicators compared to individual lipid markers for many diseases, like diabetes, and metabolic syndrome [13,27-29]. Thus, the NHHR was proposed to encompass all information related to pro-atherosclerotic and anti-atherosclerotic lipid particles, representing the balance between lipoproteins [10,30]. Previous studies have also highlighted the higher clinical utility of NHHR. Studies have demonstrated that NHHR significantly surpasses traditional lipid parameters in the assessment of atherosclerosis [11]. Similarly, in the realm of metabolic disorders, relevant experiment result indicated that NHHR has commendable diagnostic performance in identifying diabetes, metabolic syndrome, and insulin resistance, outperforming lipid indictors like non-HDL-c, LDL-c [13,15]. Notably, cohort research have confirmed a close relation between dyslipidemia and the incident of hyperuricemia [31-33]. Hence, the therapy of hyperuricemia could have clinical value. Nevertheless, there has been limited research on the correlation between lipid ratios and hyperuricemia, making it unclear whether NHHR can serve as a valuable marker for hyperuricemia. This study found that NHHR demonstrates superior diagnostic predictive value for hyperuricemia compared to the individual measurements of HDL-c. Further RCS analysis indicates a nonlinear, inverse U-shaped association in NHHR and hyperuricemia occurrence, in

line with a previous research on the non-U-shaped association in NHHR and suicidal ideation [34]. This study extends the use of lipid ratios and fills the gaps in former research, suggesting that NHHR might be a promising marker for predicting hyperuricemia. Additionally, as mentioned above, dyslipidemia is closely related to hyperuricemia, and controlling lipid levels might aid in the prevention and treatment of hyperuricemia. Whether to consider NHHR as a novel target for lipid management might require further investigation. Finally, despite the results from subgroup analysis and interaction tests, no specific populations were identified. However, other groups, such as non-white older female populations, are also worth further analysis.

Several plausible factors, like IR, oxidative stress, inflammation, application of decreasing lipid medications, might contribute to unveiling the link between dyslipidemia and hyperuricemia. Insulin resistance, a common factor behind both conditions, impairs the body's ability to use insulin efficiently, leading to disrupted lipid metabolism and increased uric acid production [35-36]. Additionally, an imbalance between free radicals and antioxidants causes oxidative stress, resulting in lipid peroxidation and uric acid accumulation [37-38]. Chronic inflammation, often seen in individuals with dyslipidemia, can also stimulate uric acid synthesis and impair its excretion [39]. Additionally, researchers have discovered that patients with hyperlipidemia and hyperuricemia share similar dietary habits, such as high-fat food intake [40]. Interestingly, lipid-lowering medications can also alter patients' SUA levels [41].

5. Study strengths and limitations

This study, utilizing sophisticated sampling weights, represents the demographic distribution of the United States. However, there exist also some deficiency to present research. First, the use of a cross-sectional design prevents us from inferring causality; prospective cohort studies and intervention trials are essential. Second, this study did not account for the application of uric acid-lowering medications, as well as common

metabolic diseases like NAFLD, which might lead to a bias in the results. Lastly, since the sample is drawn from the US population, the reliability of conclusion requires to be further proved.

6. Conclusion

In a study conducted among adults aged ≥20 years, NHHR is associated with hyperuricemia. Addressing lipid management through NHHR could assist in evaluating and preventing, as well as treating hyperuricemia.

Acknowledgments

We should acknowledge all research objects and the support from Jiangsu University and Xuzhou Medical University.

Authorship contributions

Z.W and M.W. wrote the manuscript. R.D. and F.T. prepared tables and figures. M.X. and T.G. provided critical feedback and revised the manuscript. Q.Y. supervised the entire project. All authors approved the final version .

Funding

This research has obtain support from the Sci &Tech Project of Changzhou Health Commission (WZ202226), the Kunshan key R & D program (KS2201), and the Guang Ren Foundation Research Project of Affiliated Hospital of Jiangsu University (KRY-YN2022017).

Data availability

The data is sourced from the NHANES database, which is a free resource (https://www.cdc.gov/nchs/nhanes).

Compliance with ethical standards

Conflict of Interest: there are no competing interests.

Ethical Approval: This research involving human participants were reviewed and approved by the Ethics Review Board

Informed Consent: The research objects were informed consent .

References

1. Ndrepepa G (2018) Uric acid and cardiovascular disease. Clin Chim Acta 484150-163. https://doi.org/10.1016/j.cca.2018.05.046
2. Zhu Y, Pandya BJ, Choi HK (2012) Comorbidities of gout and hyperuricemia in the US general population: NHANES 2007-2008. Am J Med 125(7): 679-687.e1. https://doi.org/10.1016/j.amjmed.2011.09.033
3. Facchini F, Chen YD, Hollenbeck CB, Reaven GM (1991) Relationship between resistance to insulin-mediated glucose uptake, urinary uric acid clearance, and plasma uric acid concentration. Jama 266(21): 3008-11.
4. Crawley WT, Jungels CG, Stenmark KR, Fini MA (2022) U-shaped association of uric acid to overall-cause mortality and its impact on clinical management of hyperuricemia. Redox Biol 51102271. https://doi.org/10.1016/j.redox.2022.102271
5. Danve A, Sehra ST, Neogi T (2021) Role of diet in hyperuricemia and gout. Best Pract Res Clin Rheumatol 35(4): 101723. https://doi.org/10.1016/j.berh.2021.101723
6. Dehlin M, Jacobsson L, Roddy E (2020) Global epidemiology of gout: prevalence, incidence, treatment patterns and risk factors. Nat Rev Rheumatol 16(7): 380-390. https://doi.org/10.1038/s41584-020-0441-1
7. Hodkinson A, Tsimpida D, Kontopantelis E, Rutter MK, Mamas MA, Panagioti M (2022) Comparative effectiveness of statins on non-high density lipoprotein cholesterol in people with diabetes and at risk of cardiovascular disease: systematic review and network meta-analysis. Bmj 376e067731. https://doi.org/10.1136/bmj-2021-067731
8. Blaha MJ, Blumenthal RS, Brinton EA, Jacobson TA (2008) The importance of non-HDL cholesterol reporting in lipid management. J Clin Lipidol 2(4): 267-73. https://doi.org/10.1016/j.jacl.2008.06.013
9. Di Bartolo BA, Cartland SP, Genner S, Manuneedhi Cholan P, Vellozzi M, Rye KA, Kavurma MM (2021) HDL Improves Cholesterol and Glucose Homeostasis and Reduces Atherosclerosis in Diabetes-Associated

Atherosclerosis. J Diabetes Res 20216668506. https://doi.org/10.1155/2021/6668506

10. Wang A, Li Y, Zhou L, Liu K, Li S, Zong C, Song B, Gao Y, Li Y, Tian C, Xing Y, Xu Y, Wang L (2022) Non-HDL-C/HDL-C ratio is associated with carotid plaque stability in general population: A cross-sectional study. Front Neurol 13875134. https://doi.org/10.3389/fneur.2022.875134

11. Zhu L, Lu Z, Zhu L, Ouyang X, Yang Y, He W, Feng Y, Yi F, Song Y (2015) Lipoprotein ratios are better than conventional lipid parameters in predicting coronary heart disease in Chinese Han people. Kardiol Pol 73(10): 931-8. https://doi.org/10.5603/KP.a2015.0086

12. Kouvari M, Panagiotakos DB, Chrysohoou C, Georgousopoulou EN, Tousoulis D, Pitsavos AC (2020) Sex-Related Differences of the Effect of Lipoproteins and Apolipoproteins on 10-Year Cardiovascular Disease Risk; Insights from the ATTICA Study (2002-2012). Molecules 25(7). https://doi.org/10.3390/molecules25071506

13. Sheng G, Liu D, Kuang M, Zhong Y, Zhang S, Zou Y (2022) Utility of Non-High-Density Lipoprotein Cholesterol to High-Density Lipoprotein Cholesterol Ratio in Evaluating Incident Diabetes Risk. Diabetes Metab Syndr Obes 151677-1686. https://doi.org/10.2147/dmso.S355980

14. Wang D, Wang L, Wang Z, Chen S, Ni Y, Jiang D (2018) Higher non-HDL-cholesterol to HDL-cholesterol ratio linked with increased nonalcoholic steatohepatitis. Lipids Health Dis 17(1): 67. https://doi.org/10.1186/s12944-018-0720-x

15. Kim SW, Jee JH, Kim HJ, Jin SM, Suh S, Bae JC, Kim SW, Chung JH, Min YK, Lee MS, Lee MK, Kim KW, Kim JH (2013) Non-HDL-cholesterol/HDL-cholesterol is a better predictor of metabolic syndrome and insulin resistance than apolipoprotein B/apolipoprotein A1. Int J Cardiol 168(3): 2678-83. https://doi.org/10.1016/j.ijcard.2013.03.027

16. Fang Y, Mei W, Wang C, Ren X, Hu J, Su F, Cao L, Tavengana G, Jiang M, Wu H, Wen Y (2024) Dyslipidemia and hyperuricemia: a cross-sectional study of residents in Wuhu, China. BMC Endocr Disord 24(1): 2. https://doi.org/10.1186/s12902-023-01528-7

17. Basnet TB, Du S, Feng R, Gao J, Gong J, Ye W (2023) Fatty liver mediates the association of hyperuricemia with prediabetes and diabetes: a weighting-based mediation analysis. Front Endocrinol (Lausanne) 141133515. https://doi.org/10.3389/fendo.2023.1133515

18. Hoffman HJ, Rawal S, Li CM, Duffy VB (2016) New chemosensory component in the U.S. National Health and Nutrition Examination Survey

(NHANES): first-year results for measured olfactory dysfunction. Rev Endocr Metab Disord 17(2): 221-40. https://doi.org/10.1007/s11154-016-9364-1

19. Hu H, Fukunaga A, Yokoya T, Nakagawa T, Honda T, Yamamoto S, Okazaki H, Miyamoto T, Sasaki N, Ogasawara T, Gonmori N, Yamamoto K, Hori A, Tomita K, Nagahama S, Konishi M, Katayama N, Morioka H, Kabe I, Mizoue T, Dohi S (2022) Non-High-Density Lipoprotein Cholesterol and Risk of Cardiovascular Disease: The Japan Epidemiology Collaboration on Occupational Health Study. J Atheroscler Thromb 29(9): 1295-1306. https://doi.org/10.5551/jat.63118

20. Xu J, Peng H, Ma Q, Zhou X, Xu W, Huang L, Hu J, Zhang Y (2014) Associations of non-high density lipoprotein cholesterol and traditional blood lipid profiles with hyperuricemia among middle-aged and elderly Chinese people: a community-based cross-sectional study. Lipids Health Dis 13117. https://doi.org/10.1186/1476-511x-13-117

21. Feig DI, Kang DH, Johnson RJ (2008) Uric acid and cardiovascular risk. N Engl J Med 359(17): 1811-21. https://doi.org/10.1056/NEJMra0800885

22. Levey AS, Stevens LA, Schmid CH, Zhang YL, Castro AF, 3rd, Feldman HI, Kusek JW, Eggers P, Van Lente F, Greene T, Coresh J (2009) A new equation to estimate glomerular filtration rate. Ann Intern Med 150(9): 604-12. https://doi.org/10.7326/0003-4819-150-9-200905050-00006

23. Navarese EP, Robinson JG, Kowalewski M, Kolodziejczak M, Andreotti F, Bliden K, Tantry U, Kubica J, Raggi P, Gurbel PA (2018) Association Between Baseline LDL-C Level and Total and Cardiovascular Mortality After LDL-C Lowering: A Systematic Review and Meta-analysis. Jama 319(15): 1566-1579. https://doi.org/10.1001/jama.2018.2525

24. Raja V, Aguiar C, Alsayed N, Chibber YS, ElBadawi H, Ezhov M, Hermans MP, Pandey RC, Ray KK, Tokgözoglu L, Zambon A, Berrou JP, Farnier M (2023) Non-HDL-cholesterol in dyslipidemia: Review of the state-of-the-art literature and outlook. Atherosclerosis 383117312. https://doi.org/10.1016/j.atherosclerosis.2023.117312

25. Yu Y, Lan T, Wang D, Fang W, Tao Y, Li M, Huang X, Zhou W, Wang T, Zhu L, Bao H, Cheng X (2021) The association of lipid ratios with hyperuricemia in a rural Chinese hypertensive population. Lipids Health Dis 20(1): 121. https://doi.org/10.1186/s12944-021-01556-z

26. Wang HP, Xu YY, Xu BL, Lu J, Xia J, Shen T, Fang J, Lei T (2023) Correlation Between Abdominal Fat Distribution and Serum Uric Acid in Patients Recently Diagnosed with Type 2 Diabetes. Diabetes Metab Syndr Obes 163751-3762. https://doi.org/10.2147/dmso.S430235

27. Chen Y, Chang Z, Liu Y, Zhao Y, Fu J, Zhang Y, Liu Y, Fan Z (2022) Triglyceride to high-density lipoprotein cholesterol ratio and cardiovascular events in the general population: A systematic review and meta-analysis of cohort studies. Nutr Metab Cardiovasc Dis 32(2): 318-329. https://doi.org/10.1016/j.numecd.2021.11.005

28. Drexel H, Larcher B, Mader A, Vonbank A, Heinzle CF, Moser B, Zanolin-Purin D, Saely CH (2021) The LDL-C/ApoB ratio predicts major cardiovascular events in patients with established atherosclerotic cardiovascular disease. Atherosclerosis 32944-49. https://doi.org/10.1016/j.atherosclerosis.2021.05.010

29. Zhang X, Zhang X, Li X, Feng J, Chen X (2019) Association of metabolic syndrome with atherogenic index of plasma in an urban Chinese population: A 15-year prospective study. Nutr Metab Cardiovasc Dis 29(11): 1214-1219. https://doi.org/10.1016/j.numecd.2019.07.006

30. Qi X, Wang S, Huang Q, Chen X, Qiu L, Ouyang K, Chen Y (2024) The association between non-high-density lipoprotein cholesterol to high-density lipoprotein cholesterol ratio (NHHR) and risk of depression among US adults: A cross-sectional NHANES study. J Affect Disord 344451-457. https://doi.org/10.1016/j.jad.2023.10.064

31. Zhang Y, Zhang M, Yu X, Wei F, Chen C, Zhang K, Feng S, Wang Y, Li WD (2020) Association of hypertension and hypertriglyceridemia on incident hyperuricemia: an 8-year prospective cohort study. J Transl Med 18(1): 409. https://doi.org/10.1186/s12967-020-02590-8

32. Xu Y, Dong H, Zhang B, Zhang J, Ma Q, Sun H (2022) Association between dyslipidaemia and the risk of hyperuricaemia: a six-year longitudinal cohort study of elderly individuals in China. Ann Med 54(1): 2402-2410. https://doi.org/10.1080/07853890.2022.2118368

33. He H, Wang S, Xu T, Liu W, Li Y, Lu G, Tu R (2023) Sex-related differences in the hypertriglyceridemic-waist phenotype in association with hyperuricemia: a longitudinal cohort study. Lipids Health Dis 22(1): 38. https://doi.org/10.1186/s12944-023-01795-2

34. Qing G, Deng W, Zhou Y, Zheng L, Wang Y, Wei B (2024) The association between non-high-density lipoprotein cholesterol to high-density lipoprotein cholesterol ratio (NHHR) and suicidal ideation in adults: a population-based study in the United States. Lipids Health Dis 23(1): 17. https://doi.org/10.1186/s12944-024-02012-4

35. Vuorinen-Markkola H, Yki-Järvinen H (1994) Hyperuricemia and insulin resistance. J Clin Endocrinol Metab 78(1): 25-9. https://doi.org/10.1210/jcem.78.1.8288709

36. Smith DA (2007) Treatment of the dyslipidemia of insulin resistance. Med Clin North Am 91(6): 1185-210, x. https://doi.org/10.1016/j.mcna.2007.06.008

37. Rizzo M, Kotur-Stevuljevic J, Berneis K, Spinas G, Rini GB, Jelic-Ivanovic Z, Spasojevic-Kalimanovska V, Vekic J (2009) Atherogenic dyslipidemia and oxidative stress: a new look. Transl Res 153(5): 217-23. https://doi.org/10.1016/j.trsl.2009.01.008

38. Gherghina ME, Peride I, Tiglis M, Neagu TP, Niculae A, Checherita IA (2022) Uric Acid and Oxidative Stress-Relationship with Cardiovascular, Metabolic, and Renal Impairment. Int J Mol Sci 23(6). https://doi.org/10.3390/ijms23063188

39. Al Shanableh Y, Hussein YY, Saidwali AH, Al-Mohannadi M, Aljalham B, Nurulhoque H, Robelah F, Al-Mansoori A, Zughaier SM (2022) Prevalence of asymptomatic hyperuricemia and its association with prediabetes, dyslipidemia and subclinical inflammation markers among young healthy adults in Qatar. BMC Endocr Disord 22(1): 21. https://doi.org/10.1186/s12902-022-00937-4

40. Conen D, Wietlisbach V, Bovet P, Shamlaye C, Riesen W, Paccaud F, Burnier M (2004) Prevalence of hyperuricemia and relation of serum uric acid with cardiovascular risk factors in a developing country. BMC Public Health 49. https://doi.org/10.1186/1471-2458-4-9

41. Deedwania PC, Stone PH, Fayyad RS, Laskey RE, Wilson DJ (2015) Improvement in Renal Function and Reduction in Serum Uric Acid with Intensive Statin Therapy in Older Patients: A Post Hoc Analysis of the SAGE Trial. Drugs Aging 32(12): 1055-65. https://doi.org/10.1007/s40266-015-0328-z

Table 1 Baseline characteristics of study population in NHANES from 2007 to 2018, weighted.

Table 2 Baseline characteristics of study population according to the quartiles of NHHR, weighted.

Table 3 Logistic regression analysis results of NHHR and hyperuricemia.

Table 4 Linear regression analysis results of NHHR and SUA.

Table 5 Threshold effect analysis of NHHR on hyperuricemia using a two-piecewise linear regression model.

Figure 1 DCA results

Figure 2 ROC results

Figure 3 Subgroup analyses

Figure 4 The results of RCS analysis. (Adjusted for age, gender, and race, annual household income, education level, smokers, diabetes, hypertension, SBP, DBP, BMI, WC, HbA1c, ALT, AST, GGT, TG, Scr, and eGFR.)

CA4-00237.docx

ORIGINALITY REPORT

20%	16%	17%	2%
SIMILARITY INDEX	INTERNET SOURCES	PUBLICATIONS	STUDENT PAPERS

PRIMARY SOURCES

1	www.frontiersin.org<br>Internet Source	5%
2	Zhaoxiang Wang, Menghuan Wu, Han Yan, Shao Zhong, Ruijun Xu, Zhiyong Zhao, Qichao Yang. "Association Between Remnant Cholesterol and Risk of Hyperuricemia: A Cross-Sectional Study", Hormone and Metabolic Research, 2024<br>Publication	3%
3	link.springer.com<br>Internet Source	1%
4	www.researchsquare.com<br>Internet Source	1%
5	lipidworld.biomedcentral.com<br>Internet Source	1%
6	www.jstage.jst.go.jp<br>Internet Source	1%
7	www.sochob.cl<br>Internet Source	1%

8	www.mdpi.com<br>Internet Source	1%
9	Minkook Son, Jeongkuk Seo, Sung Yang. "Association between dyslipidemia and serum uric acid levels in Korean adults: Korea National Health and Nutrition Examination Survey 2016-2017", PLOS ONE, 2020<br>Publication	1%
10	mdpi-res.com<br>Internet Source	1%
11	Takeshi Shimizu, Akiomi Yoshihisa, Yuki Kanno, Mai Takiguchi et al. "Relationship of hyperuricemia with mortality in heart failure patients with preserved ejection fraction", American Journal of Physiology-Heart and Circulatory Physiology, 2015<br>Publication	1%
12	Meng Zhu, Lingjuan Jia, Xiangfeng Tian, Yongsheng Zhang. "Association between cholelithiasis and frailty index in US adults: a population-based epidemiological study", Research Square Platform LLC, 2024<br>Publication	<1%
13	Zhaoxiang Wang, Menghuan Wu, Tao Pan, Xuelin Zhao, Li Zhang, Fengyan Tang, Ying Pan, Bing Lu, Shao Zhong, Song Bai. "Impaired sensitivity to thyroid hormones is	<1%

associated with albuminuria in the euthyroid population: results from NHANES", Hormones, 2024
Publication

14	www.eventscribe.net<br>Internet Source	<1 %
15	Yuanyuan He, Zhu Li, Lu Yu, Yijia Liu, Lin Li, Rongrong Yang, Xianliang Wang, Shan Gao, Chunquan Yu. "Association between the atherogenic index of plasma and carotid artery plaques in patients with coronary heart disease in different glucose metabolism states: an RCSCD-TCM study in Tianjin, China", Endocrine, 2023<br>Publication	<1 %
16	aging-us.com<br>Internet Source	<1 %
17	www.nature.com<br>Internet Source	<1 %
18	bmcendocrdisord.biomedcentral.com<br>Internet Source	<1 %
19	topsecretapiaccess.dovepress.com<br>Internet Source	<1 %
20	vdoc.pub<br>Internet Source	<1 %
21	journals.lww.com	

Internet Source

<1%

22 static.frontiersin.org
Internet Source

<1%

23 www.scilit.net
Internet Source

<1%

24 Tomonori Okamura, Kazuhisa Tsukamoto, Hidenori Arai, Yoshio Fujioka et al. "Japan Atherosclerosis Society (JAS) Guidelines for Prevention of Atherosclerotic Cardiovascular Diseases 2022", Journal of Atherosclerosis and Thrombosis, 2023
Publication

<1%

Exclude quotes	Off	Exclude matches	Off
Exclude bibliography	On		
